# Analysis of Host-Mediated Repair Mechanisms after Human CNS-Stem Cell Transplantation for Spinal Cord Injury: Correlation of Engraftment with Recovery

**DOI:** 10.1371/journal.pone.0005871

**Published:** 2009-06-11

**Authors:** Mitra J. Hooshmand, Christopher J. Sontag, Nobuko Uchida, Stan Tamaki, Aileen J. Anderson, Brian J. Cummings

**Affiliations:** 1 Department of Anatomy and Neurobiology, University of California Irvine, Irvine, California, United States of America; 2 StemCells, Inc., Palo Alto, California, United States of America; 3 Department of Physical Medicine and Rehabilitation, Reeve-Irvine Research Center, University of California Irvine, Irvine, California, United States of America; University of Auckland, New Zealand

## Abstract

**Background:**

Human central nervous system-stem cells grown as neurospheres (hCNS-SCns) self-renew, are multipotent, and have potential therapeutic applications following trauma to the spinal cord. We have previously shown locomotor recovery in immunodeficient mice that received a moderate contusion spinal cord injury (SCI) and hCNS-SCns transplantation 9 days post-injury (dpi). Engrafted hCNS-SCns exhibited terminal differentiation to myelinating oligodendrocytes and synapse-forming neurons. Further, selective ablation of human cells using Diphtheria toxin (DT) abolished locomotor recovery in this paradigm, suggesting integration of human cells within the mouse host as a possible mechanism for the locomotor improvement. However, the hypothesis that hCNS-SCns could alter the host microenvironment as an additional or alternative mechanism of recovery remained unexplored; we tested that hypothesis in the present study.

**Methods and Findings:**

Stereological quantification of human cells using a human-specific cytoplasmic marker demonstrated successful cell engraftment, survival, migration and limited proliferation in all hCNS-SCns transplanted animals. DT administration at 16 weeks post-transplant ablated 80.5% of hCNS-SCns. Stereological quantification for lesion volume, tissue sparing, descending serotonergic host fiber sprouting, chondroitin sulfate proteoglycan deposition, glial scarring, and angiogenesis demonstrated no evidence of host modification within the mouse spinal cord as a result of hCNS-SCns transplantation. Biochemical analyses supplemented stereological data supporting the absence of neural stem-cell mediated host repair. However, linear regression analysis of the number of engrafted hCNS-SCns vs. the number of errors on a horizontal ladder beam task revealed a strong correlation between these variables (r = −0.78, p<0.05), suggesting that survival and engraftment were directly related to a quantitative measure of recovery.

**Conclusions:**

Altogether, the data suggest that the locomotor improvements associated with hCNS-SCns transplantation were not due to modifications within the host microenvironment, supporting the hypothesis that human cell integration within the host circuitry mediates functional recovery following a 9 day delayed transplant.

## Introduction

The endogenous capacity of the spinal cord for repair and regeneration following traumatic injury is thought to be limited. Accordingly, stem cell transplantation is one potential strategy for promoting recovery of function after spinal cord injury (SCI). Rodent- and human-derived neural/glial cell populations transplanted sub-acutely after SCI have been associated with recovery of function in several studies [1]–[6]. In these studies, remyelination was suggested as the primary mechanism for the observed locomotor improvement. Two groups have found evidence for integration of transplanted human fetal/adult neural stem cells as new neurons following SCI [1], [7], potentially promoting restoration of disrupted circuitry. While all the studies above suggest cell integration by means of oligodendroglial and neuronal differentiation as potential mechanisms for recovery of function after SCI, the possibility of additional mechanisms whereby engrafted cell populations contribute to endogenous repair within the host microenvironment remains unexplored.

Though the presumptive strategy behind transplantation of stem cell populations for SCI has been replacement via integration of myelinating oligodendrocytes or new neurons, the ability of transplanted cell populations to affect the host niche following SCI is becoming increasingly clear. Genetically modified fibroblasts, olfactory ensheathing cells (OECs), Schwann cells, and neural stem cells (NSCs) have been reported to promote host axonal regeneration [8]–[14]. Transplantation of oligodendrocyte progenitor cells (OPCs) after SCI has been shown to promote white matter sparing [15]. Similarly, implantation of a polymer scaffold containing NSCs in the contused rat cord has been reported to reduce tissue loss and glial scarring [16], and transplantation of glial-restricted progenitors (GRPs) have been shown to reduce astroglial scarring and chondroitin sulfate proteoglycan (CSPG) deposition as early as 8 days post-transplant [13]. Other studies using GRPs have also reported modifications altering the permissiveness of the post-SCI microenvironment and promotion of regeneration [17]. Understanding the alternative mechanisms by which cell-based therapies may affect SCI will be critical in understanding how transplanted cells may affect functional recovery in a clinical setting.

Previously, our laboratory investigated the potential for human CNS-stem cells isolated from brain tissue (gestational 16–20 weeks) and grown as neurospheres (hCNS-SCns) to mediate recovery after SCI [1]. Stable hCNS-SCns lines were isolated using fluorescence-activated cell sorting (FACS) to identify a CD133^+^ and CD24^−/lo^ population [18], which enriches the number of cells in the population with neurosphere-initiating capacity approximately 2000-fold. Cells derived in this manner retain multipotency and generate neurons, astrocytes, and oligodendrocytes *in vitro* and *in vivo* [18], [19]. We have previously shown locomotor recovery in immunodeficient Non-Obese Diabetic-*severe combined immunodeficient* (NOD-*scid*) mice that received either a 50 or 60 kilodyne (kd) contusion SCI, followed by hCNS-SCns transplantation 9 days post-injury (dpi) [1]. Critically, NOD-*scid* mice enable xenogeneic transplantation without rejection/immunosuppressant drug administration, and allow achievement of 100% engraftment not seen in other models. Engrafted hCNS-SCns exhibited terminal differentiation into myelinating oligodendrocytes and synapse-forming neurons. Further, ablation of human cells using Diphtheria toxin (DT) abolished recovery in this paradigm, suggesting that cell survival is required for maintaining locomotor improvement and that integration of human cells within the mouse host is one possible mechanism for the observed recovery. However, the hypothesis that hCNS-SCns could also alter the host microenvironment as an additional mechanism was not investigated.

The current study is a stereological analysis of hCNS-SCns engraftment and the efficacy of DT ablation not previously published, in the 60 kd cohort, and additionally uses stereological analyses to test the alternative hypothesis that 9 day delayed hCNS-SCns transplantation modifies the local microenvironment in relationship to the following parameters: lesion volume, tissue sparing, descending serotonergic (5-HT) host fiber sprouting, NG2 deposition, GFAP glial scarring, and angiogenesis. Our results show no evidence of host modifications in association with hCNS-SCns transplantation. Further, biochemical analyses supplement stereological data and demonstrate no changes in Fibronectin, Versican, GFAP, NG2, and PE-CAM1 protein expression at 2 weeks post-transplant. Linear regression analysis of the number of engrafted hCNS-SCns versus the number of errors on a horizontal ladder beam task reveal a correlation between these variables, suggesting that survival/successful engraftment of hCNS-SCns is directly related to a quantitative measure of recovery. Altogether, our data suggest that integration of transplanted hCNS-SCns within the host circuitry mediates locomotor recovery in the 9 day delayed transplantation paradigm, and cell engraftment is unlikely to be associated with host niche modifications.

## Materials and Methods

### Ethics statement

All animal housing conditions, surgical procedures, and postoperative care was conducted according to the Institutional Animal Care and Use Committee (IACUC) guidelines at the University of California, Irvine.

### Contusion injuries

Female NOD-*scid* mice (10 weeks old; StemCells, Inc., Palo Alto, CA) were anesthetized with Avertin (0.5 ml/20 g tribromo-ethanol) intraperitoneally (i.p.), and received a laminectomy at vertebral level thoracic 9 (T9) using a surgical microscope. Animals (n = 65) received 60 kd (1 dyne = 10 µN) contusion injuries using the Infinite Horizon (IH) Imapctor (Precision Systems and Instrumentation. Lexington, KY). This 60 kd contusion injury resulted in average 2dpi open-field scores of 1.4±0.6 and 5.2±1.9 (S.D.) on the BMS and BBB scales, respectively. Average open-field locomotor scores at 7dpi were 3.1±1.0 and 8.3±2.2 on the BMS and BBB scales, respectively. Immediately after injury, the muscle was closed with 5–0 chromic gut sutures and the skin closed with wound clips. Animals received lactated ringers (50 ml/kg) sub-cutaneously (s.c.) immediately after surgery and for 3–5 days post surgery, and Buprenorphine (0.5 mg/kg s.c.) immediately after injury and for 2 days thereafter. Due to the immunodeficient nature of NOD-*scid* mice, and to avoid the possibility of bladder infections, antibiotics were administered daily throughout the duration of the study, rotating the use of Baytril, Ciproflaxacin, and Ampicillin (2.5 mg/kg s.c., dose for all drugs) every two weeks.

### Cell transplantation

Four mice were excluded at the time of initial SCI surgery due to unilateral bruising or abnormal force/displacement curves. Seven days post-SCI, the remaining 61 animals were behaviorally assessed using the open-field Basso, Beattie, and Bresnahan (BBB) and Basso Mouse Scale (BMS) rating scales to confirm contusion injury severity. Based on pre-transplantation open-field scores, animals were randomly assigned to receive either hCNS-SCns (average group scores of 3.15±0.65 and 10.06±0.89, BMS and BBB, respectively), human fibroblasts (hFb) (average group scores of 3.09±0.71 and 9.32±2.12, BMS and BBB, respectively), or vehicle injections (average group scores of 2.89±0.92 and 9.78±1.69, BMS and BBB, respectively); this procedure ensured all groups were composed of a set of animals in which the pre-transplantation mean and variance in scores (i.e. standard deviations) were equivalent.

**Figure 1:**
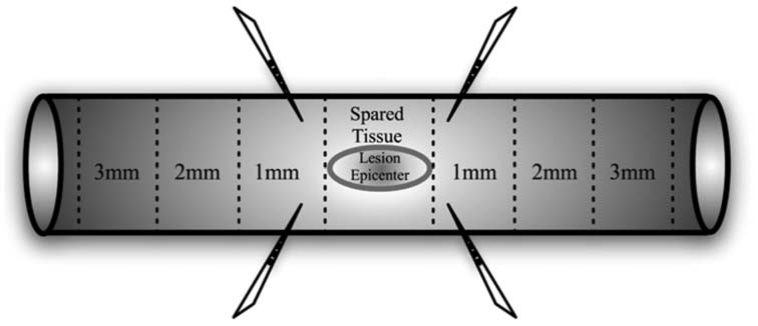
Schematic of spinal cord illustrates regions of stereological quantification for human cell numbers. Illustration depicts the five regions (lesion epicenter, spared tissue, 1 mm rostral and caudal, 2 mm rostral and caudal, 3 mm rostral and caudal) in which stereological quantification of hCNS-SCns and hFb counts were performed. Sites of cell transplantation in the two regions rostral and caudal to the lesion epicenter are also shown.

Long-term human neurosphere cultures isolated from brain tissue (gestational 16–20 weeks) have been described previously [18]. Briefly, FACS-sorted single cell suspensions were cultured in neurosphere initiation medium consisting of Ex-Vivo 15 medium with N2 supplement, FGF, EGF, LIF, neural survival factor-1, and NAC. Cultures were fed weekly and passaged every 2–3 weeks [1], [18]. Human fibroblasts were isolated from fetal liver and grown in Isocove's modified Dulbecco's medium containing 10% FBS, and passaged upon confluence [1]. On the day of transplantation, neurospheres and confluent hFb cultures were dissociated into a single-cell suspension and concentrated to a final density of 75,000 hCNS-SCns/µl of injection buffer containing 50% Hank's buffered salt solution and 50% Ex-Vivo medium and 50,000 hFb/µl of injection buffer. Nine days after SCI, mice were re-anesthetized, the laminectomy site re-exposed, and the animals were secured in a spinal stereotaxic frame by clamping the T8 and T10 lateral vertebral processes. Polished siliconized beveled glass pipettes (bevel: i.d. = 70 µm, o.d. = 100–110 µm, Sutter Instruments, Novato, CA) were loaded with freshly triturated cells, which were injected using a NanoInjector system and micropositioner (WPI Instruments, Waltham, MA). Animals received four injections bilaterally 0.75 mm from midline and into the intact parenchyma adjacent to the contusion site; cells were injected in two sites at the anterior aspect of T10 and in two sites at the posterior aspect of T8 (Fig. 1). Each site received 250 nl of hCNS-SCns or hFb or vehicle (injection buffer), for a total volume of 1 µl, delivered in 50 nl puffs over 75 s, followed by a 2 min delay before withdrawal of the pipette. Previous studies in our laboratory have demonstrated that this volume does not exacerbate damage to the spinal cord.

### Behavioral Assessment

The BMS and the modified BBB rating scales [20], [21], as well as the horizontal ladderbeam task [22] were used to assess behavioral recovery. All analyses were performed by observers blinded to treatment groups. Behavioral results have been published previously [1] and that data is used only for correlative analysis in the present study.

### Human cell ablation using Diphtheria toxin

After BBB/BMS assessment 16 weeks post-transplant, a subset of mice in each group, were randomly assigned to receive two injections of Diphtheria toxin (DT) (50 µg/kg, i.p.) 24 h apart (average BBB/BMS scores prior to DT administration: hCNS-SCns 10.35±0.88/3.93±1.65; hFb 10.00±0.91/3.38±1.62; vehicle 9.42±2.49/3.58±1.44). DT was used to selectively ablate human cells, which are 10,000 fold more sensitive to DT than mouse cells [23], [24]. The remaining animals received an equal volume of saline. All animals in the study were reassessed on the BBB/BMS, and the ladderbeam task 1 week after DT or saline administration.

### Perfusions and tissue collection

All animals (n = 48) were transcardially perfused 17 weeks post-transplant. A total of 13 animals were excluded from the study by technicians blinded to experimental groups. Exclusion criteria included kyphosis, death during the transplantation procedure, death during the study (thymic lymphoma at autopsy), and illness. Thymic lymphomas occur with high frequency in NOD-*scid* mice and life span is typically limited to 8 months under specific pathogen-free conditions [25]. Mice (n = 48) were anesthetized with a lethal dose of sodium pentobarbital (100 mg/kg, i.p.) and transcardially perfused with 30 ml of PBS, followed by 100 ml of 4% paraformaldehyde. Brains and spinal cords were dissected. Spinal cord regions corresponding to T1-T5, T6-T12, and T13-L2 vertebrae were post-fixed overnight in 4% paraformaldehyde, flash frozen at −65°C in isopentane (2-methyl butane), and stored at −80°C.

### Immunostaining

The T6-T12 spinal cord regions were embedded in optimal cutting temperature compound (Tissue-Tek). 30 µm thick parasagittal sections were cut on a sliding microtome, collected in 96-well plates containing 0.1 M Tris and 0.02% Sodium Azide, and kept at 4°C until processed for immunostaining. All immunostaining procedures were conducted at room temperature. A total of five randomly selected animals were excluded from all stereological analyses and utilized solely to establish experimental parameters: these animals were used to optimize histological procedures and determine antibody titrations.

Immunostaining was performed as previously described [26]. Briefly, sections were washed in 0.1 M Tris followed by incubation in 0.3% hydrogen peroxide/methanol. After a brief 0.1% TritonX-100 wash, sections were blocked with bovine serum albumine (BSA) and normal serum from the species in which the secondary antibody was raised. Sections were then exposed overnight to the appropriate antibody (Table 1). The next day, sections were incubated with a biotin-conjugated, purified IgG secondary antibody (1∶500, Jackson Immunoresearch) pre-adsorbed against the species in which the primary was raised (See Table 1), followed by the avidin-biotinylated peroxidase complex (ABC) using the Vectastain Elite ABC kit (Vector Laboratories, USA) and prepared according to the manufacturer's recommendations. After several washes, the signal was visualized with diaminobenzidine (DAB) (Vector Laboratories, USA). Sections were mounted onto slides and allowed to dry overnight. SC121 and GFAP-stained sections were lightly counterstained with the nuclear marker, methyl green. PE-CAM1-stained sections were counterstained with Hoechst 33342 (1∶1000, Invitrogen) to identify the lesion epicenter without interference of nuclei in brightfield. All slides were dehydrated and coverslipped using Depex mounting medium.

**Table 1:** List of antibodies used for histological and stereological assessments.

Antibody	Host	Dilution	Manufacturer	Specificity
SC121	Mouse	1∶3000 (hCNS-SCns) 1∶1000 (hFb)	StemCells, Inc.	Human Cytoplasm
GFAP	Rabbit	1∶60,000	DAKO	GFAP-positive Astrocytes
NG2	Rabbit	1∶1000	Chemicon	CSPG (NG2) Deposition
5-HT	Rabbit	1∶15,000	Sigma	Serotonergic Fibers
PE-CAM1	Rat	1∶200	Pharmingen	Endothelial Cells

### Stereological analysis

An Olympus BX51 microscope with motorized stage and StereoInvestigator (Version 6.3 Software, Microbrightfield, Williston, VT) were used to stereologically estimate unbiased total cell numbers, cell migration, lesion volume, volume of spared tissue, serotonergic fiber length, NG2 and GFAP areas, and blood vessel length. Animals containing less than three sections with a visible injury epicenter (minimum number required for obtaining reliable results) were excluded from stereological analyses (Table 2). Uniform random sampling of the tissue was performed according to standard stereological principles (see details for individual parameters below). Starting sections were chosen at random and sections were spaced 180 µm apart. Sampling parameters (i.e. grid size and counting frame size) were empirically determined to arrive at low coefficients of error (CE) for each measure. CE values are summarized in Table 3. Additionally, *post-hoc* power analysis [27], [28] of stereological data collected in previous publications from our laboratory demonstrated that stereological quantification of lesion volume [29] and serotonergic fiber sprouting [30], even with a relatively small sample size, detects medium (f/d>0.5) and large effects (f/d>0.75) with adequate statistical power (Table 4), suggesting that the methodology utilized in those studies was sensitive for detection of medium and large differences [28]. In the current study, it was estimated that the detection of a small effect (f = 0.25) with high statistical power (power = 0.95), would have required a starting total sample size of 252 animals- a number that would have been neither justifiable, nor practical in an *in vivo* setting. Accordingly, we performed a compromise analysis to meet the demands of an adequate power with a given sample size and fixed effect size, to determine whether the sample sizes in the current study were sufficient to detect small differences with adequate statistical power. Using the sample sizes in the present study (a minimum of n = 29 for PE-CAM1 and a maximum of n = 42 for GFAP scar, spared tissue, and raphespinal fibers), a small effect of f = 0.25 could have been detected with adequate statistical power of 0.63 at a minimum and 0.67 at a maximum–a power that is comparable to our previously published data where statistically significant differences were detected between groups. Thus, the study design and the stereoglocial quantification techniques utilized in the present study would be predicted to have sufficient statistical power to detect even small differences between groups with adequate power.

**Table 2:** Number of animals analyzed for individual markers using StereoInvestigator.

Group	Human Cell #		Lesion Volume		Spared Tissue		Raphespinal Fibers		GFAP Scar		NG2 Proteoglycan		Angiogenesis	
	Saline	DT	Saline	DT	Saline	DT	Saline	DT	Saline	DT	Saline	DT	Saline	DT
**Vehicle**	0	0	7	5	7	6	7	8	7	7	7	4	4	4
**hFb**	7	6	7	6	7	6	7	5	7	6	6	6	5	5
**hCNS-SCns**	7	8	7	8	7	8	7	8	7	8	6	8	4	7

Only animals containing three or more sections in which the injury epicenters were visible were included in the analyses.

**Table 3:** Intra-animal variability in stereological analyses.

	*Parameter of Analysis*						
	hCNS-SCns/hFb Number	Lesion Volume	Spared Tissue	Raphespinal Fibers	GFAP Scar	NG2 Proteoglycan	Angiogenesis Epicenter/1 mm Regions
Mean Coefficient of Error (CE)	<0.01**/**<1 per region	0.0342	0.0281	0.1184	0.0301	0.0222	0.067**/**0.088

**Table 4:** *Post-hoc* power analysis to determine statistical power of the stereological quantifications performed in two previous publications from our laboratory.

Previous Publications	Parameter Assessed	Effect Size	Alpha Error	Number of Groups	Total Sample Size	Statistical Power
**Engesser-Cesar et al. 2007**	Reaphespinal fiber length	f = 0.671*	0.05	3	19	**0.66**
**Galvan et al. 2008**	Lesion volume	d = 1.766#	0.05	2	15	**0.69**

* Effect size, f, was determined using one-way ANOVA to compare multiple groups.

# Effect size, d, was determined using Student's t-tests to compare between two groups.

### Cell number

The Optical Fractionator probe [31], [32] was used to quantify the number of human cells. The counting areas were determined by drawing contours around five different regions (1 mm, 2 mm, 3 mm, epicenter and spared tissue) of the spinal cord using a 4× objective (Fig. 1). SC121-positive cells with visible methyl-green nuclei were counted using a 100× objective in each contour. Quantification in StereoInvestigator yielded the total number of SC121-positive cells within each region. Estimated Optical Fractionator count was calculated as the sum of cells in all five regions. While engrafted hCNS-SCns migrated extensively away from the injury epicenter, our tissue collection techniques for this experiment did not include a clear marking of the rostral/caudal ends of the spinal cord, and thus, we were unable to assess direction-specific migration of hCNS-SCns (i.e. number of cells present in the rostral vs. caudal segment of the spinal cord). Therefore, in the present study, the total number of cells in each of the regions illustrated in Fig. 1 was quantified, and the sum of cell numbers for each region and in both directions was defined as the estimated number of cells in each region.

### Fiber/vessel length

Serotonergic fiber length was quantified using the Isotropic Virtual Planes (IVP) probe of StereoInvestigator [33]. A 500 µm long contour was drawn from the caudal edge of the lesion epicenter. Fiber intersects were counted using unbiased, systematic sampling using a 60× objective with a counting frame size of 50 µm×50 µm, grid size of 130 µm×200 µm, and virtual plane separation of 7.0 µm [30]. Results yielded the estimated 5-HT fiber length within this predefined 500 µm region.

Blood vessel length was quantified using the Space Balls probe of StereoInvestigator, which allows for quantification of fibers/vessels with wide diameters as was the case with PE-CAM1-positive vessels. Contours were drawn around the injury epicenter (identified by dense Hoechst staining) as well as regions 1 mm rostral and 1 mm caudal to the edge of the lesion site. Fiber intersects with a hemisphere with a 12 µm radius were counted using a 40× objective. Grid sizes of 50 µm×50 µm and 200 µm×200 µm were used at the lesion site and 1 mm regions, respectively. Results yielded estimated blood vessel length in each region.

### Area and volume analyses

Area and volume analyses were performed using Cavalieri estimator probe in StereoInvestigator [34], [35]. Lesion volume and volume of spared tissue were performed using GFAP immunostained sections. The region devoid of GFAP was identified as the lesion epicenter and areas 1 mm rostral and 1 mm caudal to the epicenter were defined as spared tissue. A point-grid of known spacing (100 µm apart) was randomly overlayed on the section image. Points were counted using a 20× objective within the region of interest. This analysis yielded the estimated lesion volume and volume of spared tissue.

Stereological analyses for GFAP astrogliosis and NG2 deposition were performed in spinal cord sections containing a visible injury epicenter. While astrogliosis and NG2 deposition are not limited to sections containing the injury epicenter, in the absence of a visible injury epicenter, the widespread expression of both proteins along the spinal cord makes the analysis of these parameters challenging. Accordingly, stereological analyses for the GFAP scar and NG2 deposition could only be performed in sections with a visible injury epicenter where the dense plexus of astrocytes as well as the NG2 proteoglycan could be reliably identified. Immunostaining for each marker was performed in 30 µm parasagittal spinal cord sections according to the stereological principles described above (one in six sections), resulting in an average of 4 sections, per marker, that contained a visible injury epicenter. Given the limited number of sections available for the analyses, volume estimation of astrogliosis and NG2 deposition would have resulted in high variability. Therefore, stereological analyses for these two parameters are reported as a measure of area, not volume. Area sampling was performed using the Cavalieri estimator probe. GFAP-occupied scar tissue was assessed using dense GFAP staining around the site of injury. A point grid of known sampling (100 µm apart) was randomly overlayed on the section image. Points were counted using a 10× objective within the region of interest. This analysis yielded the estimated area occupied by the GFAP scar or the NG2 proteogylcan.

### Biochemical protein analysis

A separate cohort of female NOD-*scid* mice (10 weeks old; StemCells, Inc., Palo Alto, CA) received 60 kd contusion injuries (n = 6) at T9 for biochemical analysis of protein expression following hCNS-SCns transplantation. All surgical and post-operation animal care procedures were identical to the methodology described above. At 7dpi, animals were behaviorally assessed using the open-field Basso Mouse Scale (BMS) rating scale to confirm contusion injury severity, and based on pre-transplantation open-field scores, animals were randomly assigned to receive either hCNS-SCns (average BMS score of 3.58±1.4) or vehicle (average BMS score of 4.50±1.87) injections.

At 9dpi, mice were re-anesthetized, the laminectomy site re-exposed, and the animals received four injections of either hCNS-SCns (n = 3) or vehicle control (n = 3) bilaterally 0.75 mm from midline and into the intact parenchyma adjacent to the contusion site; cells were injected in two sites at the anterior aspect of T10 and in two sites at the posterior aspect of T8 (Fig. 1). All surgical and post-transplantation animal care procedures were identical to the methodology described above.

Two weeks following transplantation, mice were anesthetized with a lethal dose of Euthasol. Spinal cord tissue corresponding to T7 and T11 dorsal roots was freshly dissected, and immediately placed on dry ice and stored at −80°C. Protein extraction was performed using Western blot buffer (1 M Tris-HCl containing 1.5% DTT, 2% SDS, Glycerol, 1% protease/phosphatase inhibitors added immediately before extraction, and at a pH of 6.8). Spinal cord tissue from all animals was manually homogenized, centrifuged at 13,000 rpm, and the protein samples were standardized to a concentration of 0.20 µg/µl using a Bradford Assay. A total of 16 µg of protein was loaded onto Nupage Novex 3–8% Tris-acetate gel or 10% Nupage Bis-Tris gel, separated by SDS/PAGE, and transferred to PVDF membranes by electrophoresis. The membranes were washed in 1 M Tris-buffered saline (TBS), pH 7.2–7.4, followed by Tris-buffered saline containing 3% milk and 0.01% Tween-20 (TBST), pH 7.2–7.4, for 1 hr. Primary antibodies for each antigen at the appropriate dilutions (Table 5) were added to the membranes and incubated for 2 hrs at room temperature. The membranes were washed with TBS, blocked with TBST and incubated with the appropriate secondary antibody for 1 hr (Table 5). The blots were then washed with TBS, and blocked with TBST. In a final step, the blots were either incubated with ECL solution (GE Healthcare) and signal was detected on Hyperfilm-ECL (GE Healthcare) or the blots were incubated with Avidin-Biotin Complex for 30 minutes and signal was visualized using the VIP Peroxidase kit (Vector Laboratories). Protein levels were quantified by an individual blinded to experimental groups using ImageJ (Version 10.2) gel analyzer.

**Table 5:** List of antibodies used for biochemical protein analysis (western blot).

Antibody	Host	Dilution	Manufacturer	Secondary Antibody	Dilution	Manufacturer
Fibronectin	Rabbit	1∶500	Sigma-Aldrich	Amersham ECL Rabbit IgG, HRP-linked	1∶10,000	GE Healthcare
GFAP	Rabbit	1∶40,000	DAKO	Amersham ECL Rabbit IgG, HRP-linked	1∶10,000	GE Healthcare
NG2	Rabbit	1∶500	Chemicon	Amersham ECL Rabbit IgG, HRP-linked	1∶10,000	GE Healthcare
Versican	Rabbit	1∶400	Chemicon	Amersham ECL Rabbit IgG, HRP-linked	1∶10,000	GE Healthcare
PE-CAM1	Goat	1∶500	R&D Systems	Anti-goat IgG	1∶500	Jackson Immunoresearch

### Statistical analysis

All behavioral, histological, stereological, and biochemical analyses were performed by experimenters blinded to the experimental groups. Statistical analyses were performed using Statview (Version 5.0.1 Software) and Prism (Version 4.0a Software). Power analyses were performed using G*Power (Version 3.0.10 Software). All stereological quantifications for measures of host recovery were analyzed using one-way ANOVA to compare differences between the three groups. Biochemical protein levels (western blots) were analyzed using two-tailed Student's t-tests to compare differences between two groups. Linear regression analysis was performed with Pearson correlation, followed by the appropriate t-tests. Significance was defined as p<0.05 in all statistical analyses.

## Results

### Cell engraftment and survival in animals receiving saline at 16 weeks post-transplant

**Figure 2:**
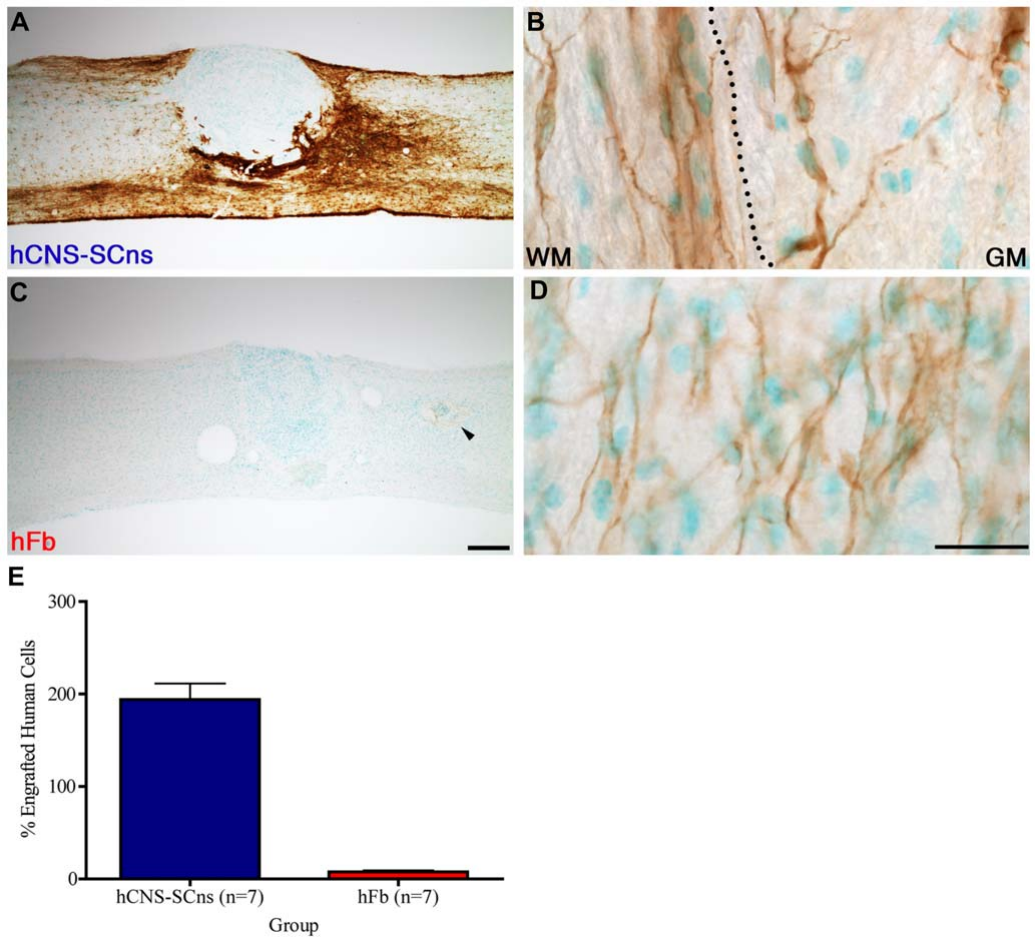
hCNS-SCns engraft, survive, and show limited proliferation in all transplanted animals; hFb engraft in all transplanted animals, but demonstrate poor survival. A: Immunostaining using a human-specific cytoplasmic marker (SC121) demonstrated that hCNS-SCns survived 17 weeks post-transplant. Brown indicates human cells visualized with DAB; Green indicates mouse and human nuclei visualized with methyl green.B: Engrafted hCNS-SCns migrated away from the injury and appeared to differentiate in a site-specific manner with oligodendroglial and neuronal morphologies in the white and grey matters, respectively. C: hFb also survived 17 weeks post-transplant, but were localized near the site of transplantation (arrowhead).D: High power image of arrowhead in (C) demonstrated the presence of hFb at the site of injection.E: At 9dpi, animals received either 75,000 hCNS-SCns or 50,000 hFb. Human cells engrafted in 100% of the animals. At 17 weeks post-transplant, an average of 194% of the initial dose of hCNS-SCns and 7.5% of the initial dose of hFb were found in each animal, suggesting limited hCNS-SCns proliferation. Bars represent group means±standard errors. Scale bars = 250 µm for A and C and 25 µm for B and D.

One of the challenges associated with any cell transplantation strategy, especially in a xenograft paradigm, is the ability to overcome the host immune response and obtain successful cell engraftment. In the present study, we used immunodeficient NOD-*scid* mice to reduce confounds of xenograft rejection, avoid immunosuppressant drugs, and enhance hCNS-SCns engraftment success. Immunostaining using the human-specific cytoplasmic marker, SC121, revealed that hCNS-SCns engrafted in 100% of the animals, and that engrafted cells survived 17 weeks post-transplant (Fig. 2A). Engrafted hCNS-SCns migrated along the spinal cord and appeared to differentiate in a site-specific manner consistent with neuronal and oligodendroglial morphologies in the grey and white matter, respectively (Fig. 2B). Confocal analysis of hCNS-SCns differentiation was not repeated in the present study but was performed in our previous publication [1], where we reported evidence for β-tubulin or APC/CC1 expression in 26.4% and 64.1% of engrafted hCNS-SCns, respectively. hFb also engrafted in 100% of the animals and survived 17 weeks post-transplant (Fig. 2C). In contrast to hCNS-SCns, engrafted hFb were highly localized to the region corresponding to the site of transplantation and exhibited limited migration (Fig. 2C and D).

Stereological quantification for the total number of engrafted cells demonstrated an average of 145,553 hCNS-SCns in comparison to the 75,000 cells originally transplanted (194% of the initial transplant dose), suggesting limited proliferation (Fig 2E). Notably, no evidence of tumorogenesis or increased mortality in the transplanted versus non-transplanted animals was observed during the total duration of the study. Addtionally, stereological quantification demonstrated an average of 3,752 hFb in comparison to the 50,000 cells originally transplanted (7.5% of the initial transplant dose), suggesting that the spinal cord niche is not a favorable environment for hFb survival (Fig. 2E).

### Cell migration in animals receiving saline at 16 weeks post-transplant

**Figure 3:**
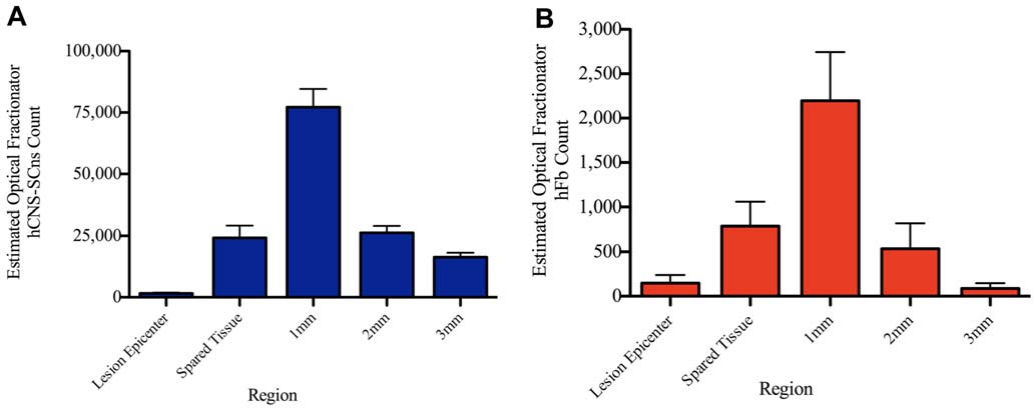
hCNS-SCns migrate rostral and caudal to the injury; hFb migrate to a lesser extent. A: Estimated number of hCNS-SCns at specific distances from the injury epicenter and along the spinal cord (shown in Fig. 1) was quantified. hCNS-SCns migrated at least 3 mm rostral and 3 mm caudal to the site of injury, but the majority of hCNS-SCns (average of 77,180±7,423) were found at regions 1 mm rostral and caudal to the lesion epicenter. B: Estimated number of hFb was quantified in the same manner as hCNS-SCns. While hFb were also found to migrate 3 mm rostral and 3 mm cadudal to the lesion site, less hFb were found away from the injury epicenter compared to hCNS-SCns. The majority of hFb were also found in regions 1 mm rostral and caudal to the injury site (average of 2,196±548) corresponding the original site of transplant. Bars represent group means±standard errors.

Effective therapeutic remyelination after SCI may only be possible if the transplanted cell population is capable of extensive migration, enabling cells to reach demyelinated and/or dysmyelinated axons above and below the primary injury. To investigate cell migration, we analyzed the number of human cells at different regions along the spinal cord (see Methods) and found that both hCNS-SCns and hFb migrated at least 3 mm, in each direction from the edge of the lesion, but significantly more hCNS-SCns were found at greater distances from the epicenter in comparison to hFb (Fig. 3A and B). hCNS-SCns were clearly observed to have migrated beyond 3 mm, however, in order to preserve stereological consistency, only cells within regions 3 mm in each direction from the injury site were quantified. The majority of both hCNS-SCns and hFb were found at regions 1 mm away from the injury epicenter (an average of 77,180±7,423 hCNS-SCns and an average of 2,196±548 hFb) corresponding to the original sites of transplantation. Few cells (an average of 1,600±276 hCNS-SCns and an average of 147±92 hFb) were recruited into the lesion site (Fig. 3A and B). Collectively, these data show that following a 9 day delayed transplantation, hCNS-SCns are capable of migration in the injured spinal cord.

### Human cell ablation using Diphtheria toxin at 16 weeks post-transplant

It is possible that transplantation of a cell population after SCI could recruit host cells capable of contributing to locomotor recovery. Indeed, one study has demonstrated that while transplanted Schwann cells stimulated host Schwann cell recruitment, the grafted cells showed limited survival at the epicenter of a contusion SCI, and represented a minority of total Schwann cells present in the cavity two weeks post-transplantation [36]. However, if recovery in a given paradigm were mediated by recruited host cells, one would predict that continued survival of the transplanted cell population would not be required to maintain behavioral improvement. In contrast to that study, our previous Diphtheria toxin (DT) ablation of engrafted human cells demonstrated that survival of engrafted hCNS-SCns was required for maintenance of functional locomotor recovery [1]. In the present study, we quantified the number of human cells surviving post-DT treatment in order to establish a numerical estimate of ablation efficacy for this technique.

**Figure 4:**
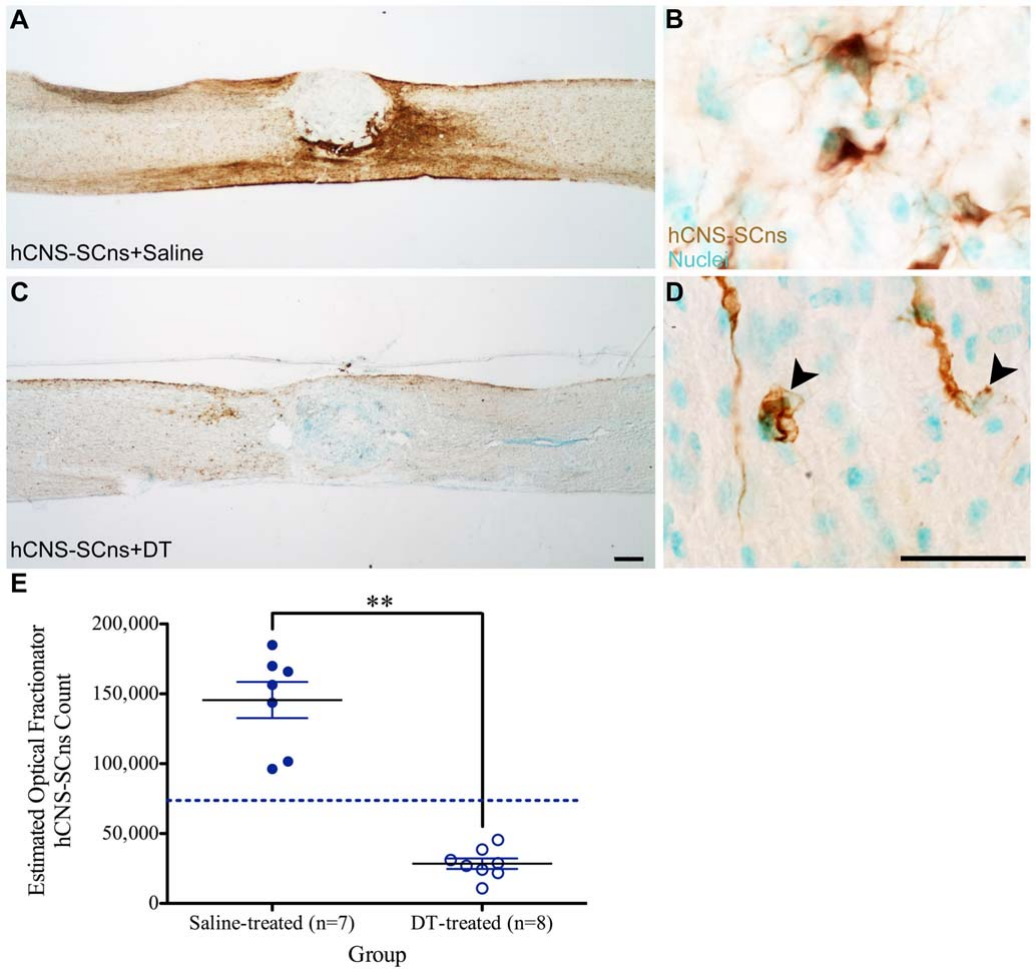
Diphtheria toxin (DT) administration at 16 weeks post-transplant ablates hCNS-SCns. A: Immunostaining using SC121 revealed cell survival in animals that received hCNS-SCns at 9dpi and saline injection at 16 weeks post-transplant. B: High power image of (A) demonstrated the presence of healthy cells with staining of cytoplasm and processes. C: Animals receiving hCNS-SCns at 9dpi and DT at 16 weeks post-transplant demonstrated a reduction in SC121 immunostaining. D: High power image of (C) demonstrated the presence of hCNS-SCns with foamy appearance and/or morphological characteristics representative of unhealthy or apoptotic/necrotic cells (arrowheads). E: Quantification for the estimated number of hCNS-SCns 17 weeks post-transplant revealed limited proliferation of hCNS-SCns in animals receiving saline at 16 weeks post-transplant. Dashed line indicates the original transplanted dose of 75,000 hCNS-SCns. DT administration resulted in a significant reduction (80.5%) in the number of hCNS-SCns. ** denotes p<0.0001, 1-tailed t-test. Means±standard errors are shown. Scale bars = 250 µm for A and C and 25 µm for B and D.

**Figure 5:**
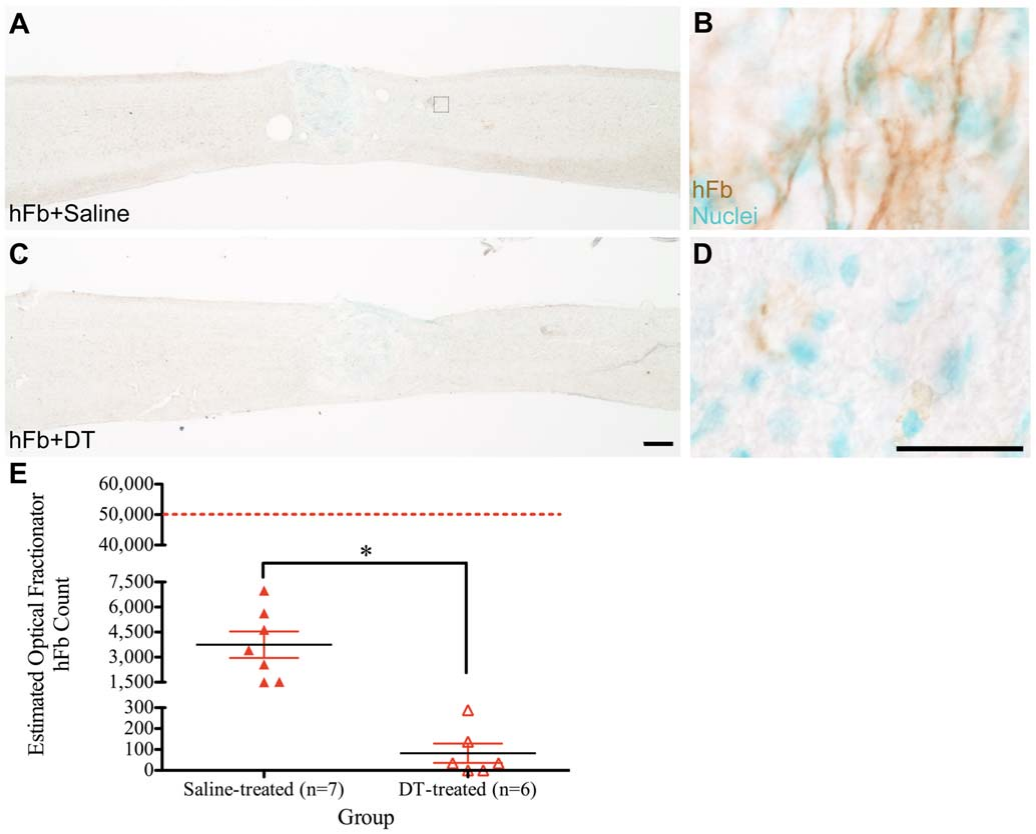
Diphtheria toxin (DT) administration at 16 weeks post-transplant ablates hFB. A: Immunostaining using SC121 illustrated limited cell engraftment/survival (inset) in animals that received hFb at 9dpi and saline injection at 16 weeks post-transplant. B: High power image of inset in (A) revealed the presence of hFb near the site of transplantation. C: Animals receiving hFb at 9dpi and DT at 16 weeks post-transplant demonstrated a reduction in SC121 immunostaining. D: High power image of (C) demonstrated the absence of SC121 immunoreactivity in animals receiving DT at 16 weeks post-transplant. E: Quantification for the estimated number of hFb in animals receiving saline at 16 weeks post-transplant revealed limited survival. Dashed line indicates the original transplanted dose of 50,000 hFb. DT administration resulted in a significant reduction in the number of hFb (97.8%) suggesting that DT ablated the transplanted human cells. * denotes p<0.0007, 1-tailed t-test. Means±standard errors are shown. Scale bars = 250 µm for A and C and 25 µm for B and D.

Histological analysis in animals receiving human cells at 9dpi and 2 injections of DT at 16 weeks post-transplant demonstrated an 80.5% reduction in the number of hCNS-SCns (Fig. 4C and D) and 97.8% reduction of hFb (Fig. 5C and D) in comparison to animals that received cells and were treated with saline at 16 weeks post-transplant (Fig. 4A–B and Fig. 5A–B). Stereological quantification of the total number of engrafted human cells within the defined region of 3 mm rostral and 3 mm caudal to the injury epicenter revealed the presence of an average of 145,553±12,940 hCNS-SCns (Fig. 4E) and an average of 3,752±790 hFb (Fig. 5E) in animals receiving saline injection at 16 weeks post-transplant. In comparison to the saline-treated animals, mice receiving DT exhibited significantly less hCNS-SCns (an average of 28,454±4,007) (Fig. 4E) and hFb (an average of 82±45) (Fig. 5E), demonstrating that DT ablated the human cells (*1-tailed t-tests: p<0.0001 for hCNS-SCns and p<0.0007 for hFb*). It is important to note that the estimated number of human cells in animals treated with DT reported here is likely to be an overestimation of intact, surviving cells. As shown in Fig. 4D, apoptotic/necrotic appearing SC121-positive cells with nuclei and a “foamy” appearance, but without processes, were detected in animals receiving DT (arrowheads). However, in order to ensure stereological consistency and reduce bias in quantification, all SC121-positive human cells that were associated with methyl green-positive nuclei were counted, whether or not they were morphologically “unhealthy.” Accordingly, a conservative but accurate estimate for the number of hCNS-SCns in the DT-treated animals is reported. Collectively, these findings extended and confirmed our previous observations that human cells are ablated by administration of DT.

### Stereological quantification for changes in the host microenvironment

While improved locomotor recovery following 9dpi hCNS-SCns transplantation was previously associated with cell integration within the host circuitry [1], the hypothesis that engrafted cells could alter the host microenvironment as an additional mechanism of recovery remained unexplored. In this study, we tested that hypothesis by selecting multiple parameters of host response that were most likely to undergo change following transplantation. In the following sections, we discuss the effect of engrafted hCNS-SCns in relationship to lesion volume, tissue sparing, serotonergic fiber sprouting/regeneration, NG2 deposition, astrogliosis, and angiogenesis. Stereological analyses of these parameters compare three groups: vehicle, hFb, and hCNS-SCns. While a subset of animals in each group received DT treatment at 16 weeks post-transplant, unbiased stereology demonstrated no significant differences in any of the parameters between DT- and saline-treated animals, suggesting that the one week administration of DT did not affect the host microenvironment. Thus, with the exception of the correlative analyses, where only saline-treated animals were analyzed, the means reported henceforth include all animals within each group (vehicle, hFb, and hCNS-SCns), both DT- and saline-treated.

### Lesion volume and tissue sparing

**Figure 6:**
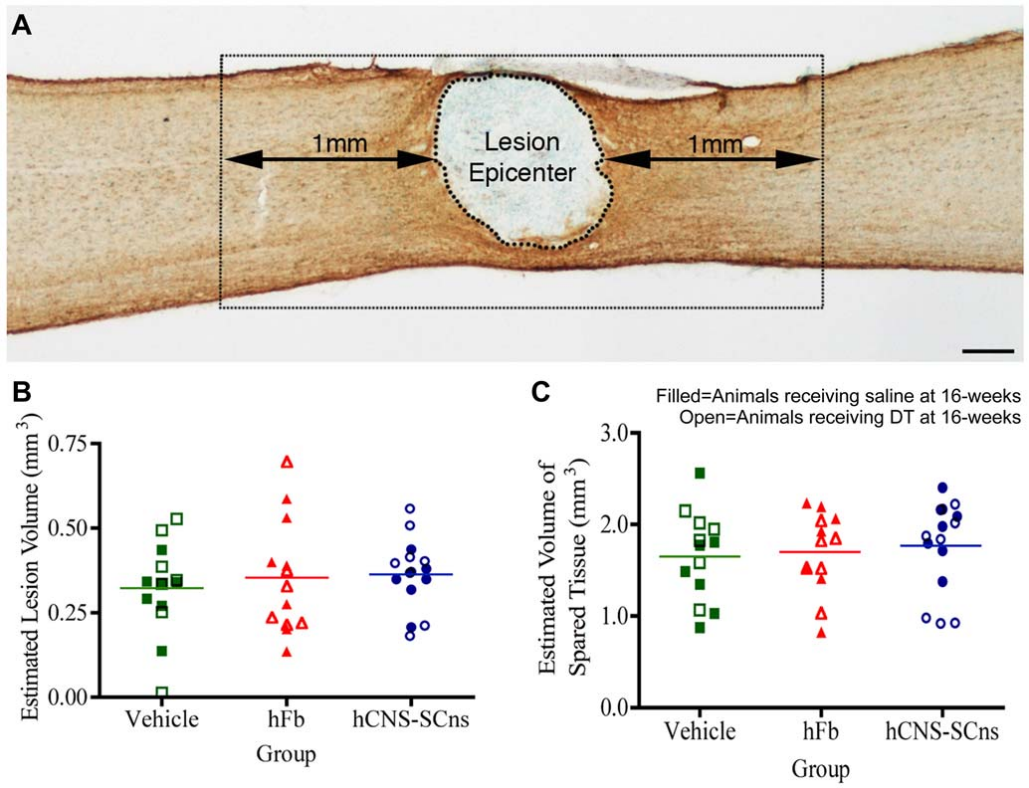
Human cell transplantation does not alter the volumes of lesion epicenter or spared tisse. A: The lesion epicenter was identified as the region devoid of GFAP immunostaining. Regions 1 mm rostral and 1 mm caudal to the border of the lesion were selected for assessment of spared tissue. B: Volume assessments were performed using the Cavalieri estimator probe of StereoInvestigator and revealed no significant differences in the estimated lesion volume between any of the groups (*One-way ANOVA: F = 0.51, p = 0.60*). C: No significant differences were found in the estimated volume of spared tissue between any of the groups (*One-way ANOVA: F = 0.20, p = 0.82*). Scale bar = 250 µm.

The environment after trauma is dynamic and cell death persists for weeks following SCI [37]–[39] resulting in an increase in lesion size and a decrease in the spared tissue around the injury. Delayed hCNS-SCns transplantation could reduce apoptosis/necrosis by providing neurotrophic support to neurons and glia [38]–[40]. Thus, we examined the potential contributions of transplanted cells to changes in total lesion size and the amount of tissue spared. To investigate the possibility that cells transplanted at 9dpi affected the ongoing evolution of the injury epicenter, estimated lesion volume was assessed using unbiased stereology. As described under Methods, the injury epicenter was defined as the area devoid of GFAP immunostaining and the volume of this region (Fig. 6A) was quantified using the Cavalieri estimator. No significant differences (*One-way ANOVA: F = 0.51, p = 0.60*) were detected between any of the groups (vehicle, hFb, and hCNS-SCns) in the estimated lesion volume (Fig. 6B).

To evaluate the amount of spared tissue proximal to the injury epicenter and transplantation sites, the volume of intact tissue was quantified in the same sections analyzed for lesion volume. Spared tissue was defined within constrained regions extending 1 mm rostral and caudal from the edge of injury epicenter, including both white and grey matters (Fig. 6A). Cavalieri sampling in this region revealed no significant differences (*One-way ANOVA*: *F = 0.20, p = 0.82*) between any of the groups in the estimated volume of spared tissue (Fig. 6C). These data suggest that neither hCNS-SCns nor hFb alter the lesion volume or volume of spared tissue.

### Raphespinal regeneration/sprouting

**Figure 7:**
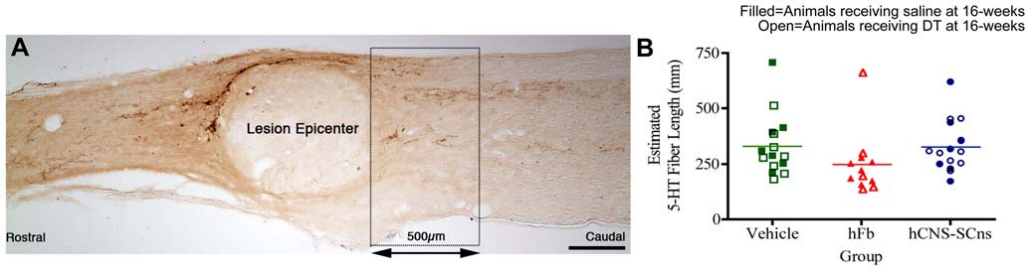
Human cell transplantation does not alter raphespinal sprouting/regeneration. A: A contour was drawn around a 500 µm long region at the caudal edge of the lesion epicenter and 5-HT fiber length in this area was quantified using the Isotropic Virtual Planes probe of StereoInvestigator. B: Stereological analysis revealed no significant differences in the estimated raphespinal fiber length between any of the groups *(One-way ANOVA: F = 1.67, p = 0.20)*. Scale bar = 250 µm.

The raphespinal tract is significantly involved in locomotor function in rodents [41]–[43]. Additionally, because few serotonergic (5-HT) cells are located within the spinal cord, the bulk of raphespinal fiber immunostaining can be considered descending [30], making it an appropriate tract for the assessment of fiber regeneration/sprouting post-SCI. Regeneration/sprouting of the 5-HT fiber tract has been previously shown in several cell transplantation models via grafting of BDNF and NT-3 producing fibroblasts into injured rat spinal cords [11], [14], [41], [44], [45]. To investigate the possibility that cells transplanted 9dpi affected regeneration/sprouting of serotonergic fibers, unbiased stereological quantification of 5-HT immunopositive fibers was performed using the Isotropic Virtual Planes probe in a 500 µm long region measured from the caudal edge of the lesion epicenter where regenerating/sprouting fibers are found after injury (Fig. 7A). Estimated fiber length was not significantly different (*One-way ANOVA*: *F = 1.67, p = 0.20*) across any of the cell-treated groups or vehicle control (Fig. 7B). This data suggests that neither hCNS-SCns nor hFb promote regeneration and/or sprouting of host serotonergic fibers.

### NG2 deposition

During the chronic phases of injury, an astroglial scar, which has been suggested to be non-permissive to axonal regeneration, forms around the site of lesion. The growth-inhibitory environment after SCI, however, is not limited to astrogliosis. A wide variety of extracellular matrix molecules in the CSPG family, including NG2, brevican, versican, and neurocan are also differentially up-regulated after injury [46]–[50], and have been shown to limit neurite outgrowth [51]–[53]. Numerous studies in rats have assessed CSPG deposition after SCI using a pan-CSPG antibody [46], [54]–[56]. Other studies performed in mice have also reported CSPG deposition post trauma using the same antibody [57]. However, due to the monoclonal nature of available anti-CSPG antibodies and our animal model (mouse), we were unsatisfied with the level of specificity with this approach (data not shown). We therefore sought to test the possible cell-mediated changes in deposition of a more specific CSPG, the NG2 proteogylcan.

**Figure 8:**
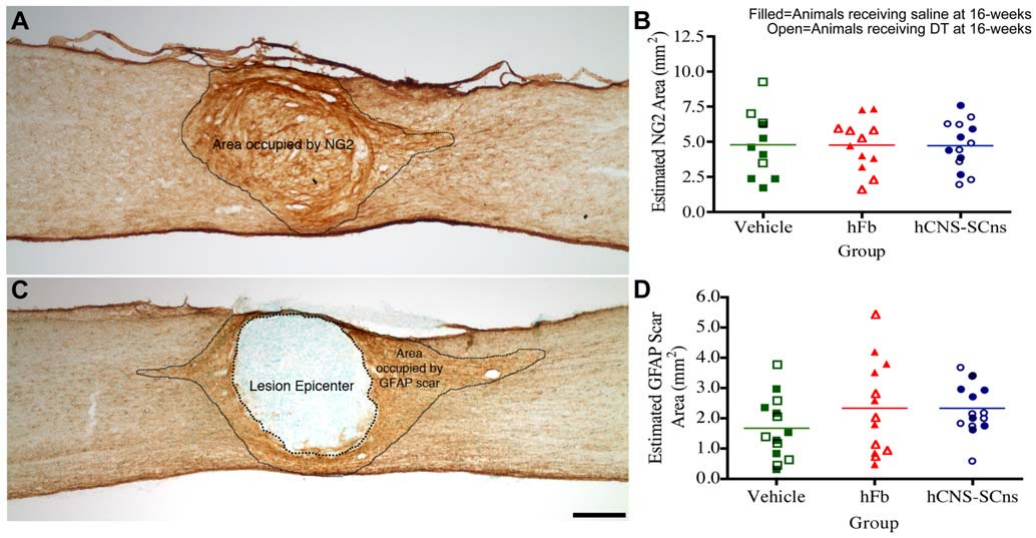
Human cell transplantation does not alter the areas of NG2 deposition or the GFAP astroglial scar. A: Estimated area occupied by the NG2 proteoglycan was analyzed using the Cavalieri estimator probe of StereoInvestigator. B: Quantification revealed no significant differences between any of the groups in the area occupied by NG2 (*One-way ANOVA: F = 0.005, p = 0.99*). C: Estimated area occupied by the GFAP scar was determined in the same manner as NG2. The lesion epicenter was not included in the estimated GFAP scar area. D: Stereological quantification exhibited no significant differences between any of the groups in the area of the GFAP astroglial scar (*One-way ANOVA: F = 1.50, p = 0.24*). Scale Bar = 250 µm for A and C.

NG2 is a major CSPG component that is elevated starting 24 hours after injury, remaining significantly above control levels for up to 6 months post-SCI, and is inhibitory to neurite outgrowth *in vitro* [58]–[60] and *in vivo* [58], [59], [61]. Previous transplantation studies have demonstrated the capacity of embryonic radial glia to reduce NG2 deposition, thus promoting axonal regeneration [54]. In order to investigate the effect of cell transplantation at 9dpi on NG2 deposition, stereological quantification of the area occupied by the NG2 proteoglycan (Fig. 8A) was performed using the Cavalieri estimator. No significant differences (*One-way ANOVA*, *F = 0.005, p = 0.99*) were found in the area occupied by the NG2 proteogylcan between any of the groups (Fig. 8B). This data suggests that neither hCNS-SCns nor hFb alter deposition of the NG2 proteoglycan following injury.

### GFAP astrogliosis

Following CNS trauma, astrocytes proliferate, hypertrophy, and extend processes, walling off the region around the injury epicenter [62]. Acutely after SCI, astrocytes may serve a beneficial role by mediating repair of the blood-brain barrier [17], [63]. In more chronic phases of injury, however, reactive astrocytes have been suggested to inhibit neurite outgrowth by operating as a chemical barrier by releasing a variety of CSPGs, as discussed above, and as a complex, three-dimensional physical barrier [52], [58], [59], [64]. While we did not observe any changes in deposition of the scar-associated NG2 proteoglycan as a result of cell transplantation, the possibility that engrafted cells modified the physical component of astrogliosis remained unclear. In this experiment, we investigated the effect of cell transplantation at 9dpi on the size of the astroglial scar. Stereological quantification for the area surrounding the injury epicenter and containing dense GFAP-positive immunostaining indicative of the glial scar (Fig. 8C) was performed using the Cavalieri estimator probe. No significant differences (*One-way ANOVA*, *F = 1.50, p = 0.24*) in the area occupied by the GFAP astroglial scar were found between any of the groups (Fig. 8D). These findings suggest that neither hCNS-SCns nor hFb alter astrogliosis when compared to vehicle control. Further, it suggests that hCNS-SCns do not contribute to the astroglial scar by recruitment of host GFAP cells.

### Angiogenesis

**Figure 9:**
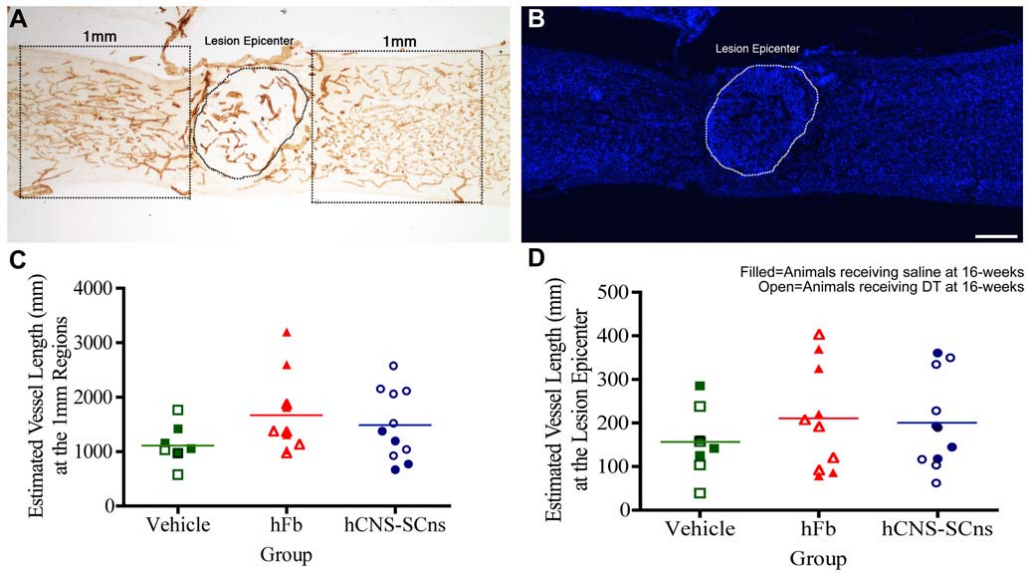
Human cell transplantation does not alter angiogenesis. A: Estimated length of blood vessels at the injury epicenter and at regions 1 mm rostral and 1 mm caudal to the lesion site were quantified using the Space Balls probe of StereoInvestigator. B: The injury epicenter was identified using DAPI immunofluorescent staining within the PE-CAM1-stained sections. C: While there was a trend toward an increase in blood vessel length at regions 1 mm rostral and caudal to the lesion, this difference was not statistically significant (*One-way ANOVA: F = 1.87, p = 0.17*). D: No significant differences in the blood vessel length were detected between the groups at the injury epicenter (*One-way ANOVA: F = 0.65, p = 0.53*). Scale Bar = 250 µm for A and B.

Spinal cord injury directly affects the permeability of the blood-brain/spinal barrier and damages the spinal cord vasculature. Application of a number of treatments following CNS trauma has been suggested to promote behavioral recovery associated with vascular remodeling [65], [66]. Notably, transplantation of OECs after a variety of PNS and CNS injury paradigms has been shown to modulate angiogenesis either directly via an increase in blood vessel density [67] or indirectly by an increase in endothelial-associated growth factors [68]. We tested the hypothesis that cell transplantation mediates spinal cord vasculature repair using immunostaining with PE-CAM1 (platelet/endothelial cell adhesion molecule). Blood vessel length was quantified using the Space Balls probe of StereoInvestigator in two regions: 1) at the injury epicenter and 2) 1 mm rostral and 1 mm caudal to the edge of the lesion (Fig. 9A and B). While there was a trend for increased blood vessel length at regions 1 mm rostral and 1 mm caudal to the injury epicenter in transplanted animals compared to control groups, this difference did not achieve statistical significance (*One-way ANOVA, F = 1.87, p = 0.17*) (Fig. 9C). Similarly, no significant differences were found at the lesion site (*One-way ANOVA, F = 0.65, p = 0.53*) (Fig. 9D). These findings suggest that neither hCNS-SCns nor hFb modify revascularization after SCI.

### Biochemical protein analyses

**Figure 10:**
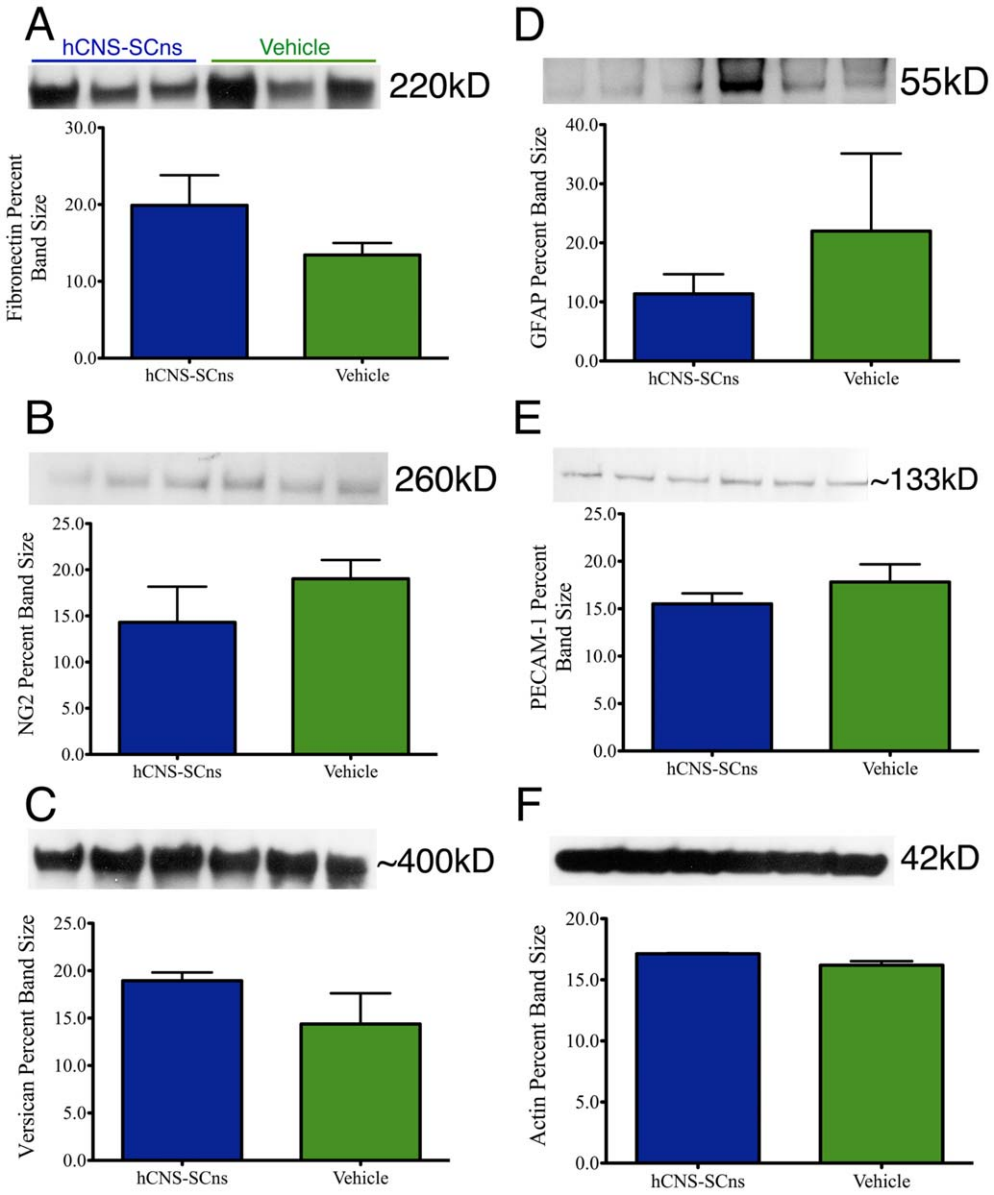
Expression levels of proteins associated with host repair remain unchanged as a result of hCNS-SCns transplantation. Biochemical analysis was performed 2 weeks post-transplant in animals that received either hCNS-SCns or vehicle control. A: No changes in Fibronectin protein expression were observed as a result of hCNS-SCns transplantation (*2-tailed t-test: p = 0.234*). B: Protein analysis of a CSPG protein, NG2, demonstrated no significant differences between transplanted animals and vehicle control (*2-tailed t-test: p = 0.477*). C: Expression levels of an additional CSPG protein, Versican, revealed no significant differences between the two groups (*2-tailed t-test: p = 0.299*). D: GFAP protein expression remained unchanged as a result of hCNS-SCns transplantation (*2-tailed t-test: p = 0.581*). E: PE-CAM1 expression levels were not altered after cell transplantation (*2-tailed t-test: p = 0.496*). F: β-actin was used as control to ensure equivalent protein loading (*2-tailed t-test: p = 0.116*).

The histological absence of significant differences in the multiple parameters of host response after hCNS-SCns transplantation was supplemented with biochemical analyses to determine protein expression levels in spinal cord-injured animals receiving either hCNS-SCns or vehicle at 9dpi. We hypothesized that the maximum potential for trophic/neuroprotective modulation of host repair mechanisms by transplanted cells would occur within the first 2 weeks following transplantation because lesion volume, tissue sparing, astroglial scar formation and proteoglycan deposition become relatively stable within 2–3 weeks following rodent contusion SCI [69]–[72]. Hence, assessment of a supplemental set of biochemical markers for host repair at this additional time point of 2 weeks post-transplantation would have both the potential to reveal any changes missed in our analyses at 4 months post-transplantation, and the potential to reveal any changes that may have occurred acutely but transiently after transplantation. Accordingly, western blot analysis to assess possible changes in protein expression levels of fibronectin, an extracellular matrix molecule present at the lesion epicenter of injured mice, revealed no significant differences (*2-tailed t-test: p = 0.234*) between animals receiving hCNS-SCns and vehicle (Fig. 10A), suggesting that hCNS-SCns transplantation does not alter fibronectin deposition at 2 weeks post-transplant. The protein expression levels of two CSPG proteins, NG2 and Versican, which have been shown to increase acutely after injury and remain upregulated for prolonged periods following SCI, also did not change (*2-tailed t-test: p = 0.477 for NG2 and p = 0.299 for Versican*) as a result of hCNS-SCns transplantation (Fig. 10B and C). Further, GFAP protein expression (Fig. 10D), an indication of glial scarring, and PE-CAM1 expression (Fig. 10E), indicative of potential angiogenesis, were not different between transplanted animals and vehicle control (*2-tailed t-test: p = 0.581 for GFAP and p = 0.496 for PE-CAM1*). Protein expression level of β-actin, which was used to ensure equivalent protein loading, demonstrated no significant differences (*2-tailed t-tests: p = 0.116*) between transplanted animals and vehicle control (Fig. 10F). Collectively, these data supplement our stereological quantification suggesting that hCNS-SCns transplantation does not alter lesion size, CSPG deposition, GFAP glial scarring, and re-vascularization.

### Correlative analyses

We have previously shown that hCNS-SCns, but not hFb, improved locomotor recovery assessed by the horizontal ladderbeam task after 9 day delayed transplantation into the injured spinal cord [1]. In the present study, we demonstrated that hCNS-SCns survived and migrated 17 weeks post-transplant and administration of DT ablated the human cells. Additionally, we found no evidence of host modification in transplanted animals, suggesting that cell transplantation does not alter endogenous repair mechanisms. However, the possibility that the *number* of engrafted cells affected behavioral and histological recovery remained unexplored. To further investigate this relationship, we performed regression analyses for the total number of engrafted human cells and behavioral/histological host recovery. All correlation analyses were performed in the subset of animals receiving human cells at 9dpi and saline at 16 weeks post-transplant (Table 2). As shown previously (Figs. 4 and 5), DT ablated 80.5% of hCNS-SCns and 97.8% hFb; for this reason, animals receiving DT were excluded from regression analyses.

**Figure 11:**
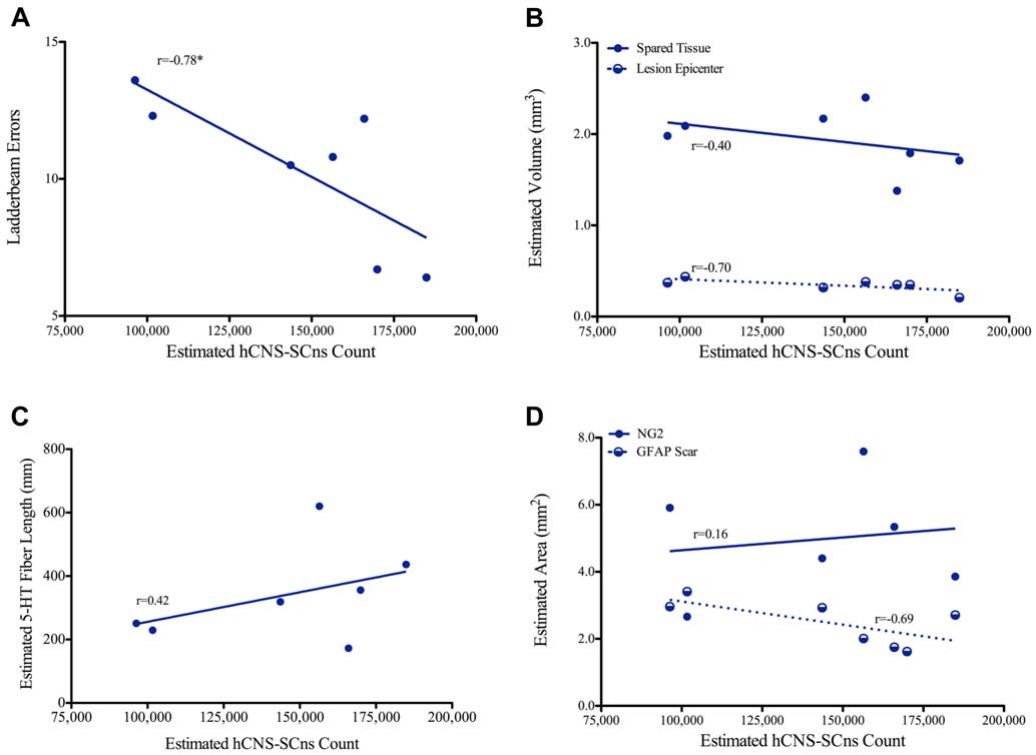
hCNS-SCns engraftment directly correlates with a quantitative measure of behavior, but not other measures of histological recovery. A: Linear regression analysis revealed a significant negative correlation between hCNS-SCns engraftment and the number of errors made on the horizontal ladderbeam task (*Pearson r = −0.78, p = 0.038, 2-tailed t-test*). B-D: Linear regression analyses for the estimated number of hCNS-SCns and other measures of host recovery revealed no significant correlations between cell engraftment and lesion volume (B) (*Pearson r = −0.70, p = 0.08, 2-tailed t-test*), volume of spared tissue (B) (*Pearson r = 0.42, p = 0.34, 2-tailed t-test*), serotonergic fiber sprouting (C) (*Pearson r = 0.16, p = 0.77, 2-tailed t-test*), NG2 area (D) (*Pearson r = 0.16, p = 0.77, 2-tailed t-test*), and area of the GFAP astroglial scar (D) (*Pearson r = −0.69, p = 0.08, 2-tailed t-test*). * denotes p<0.05.

**Figure 12:**
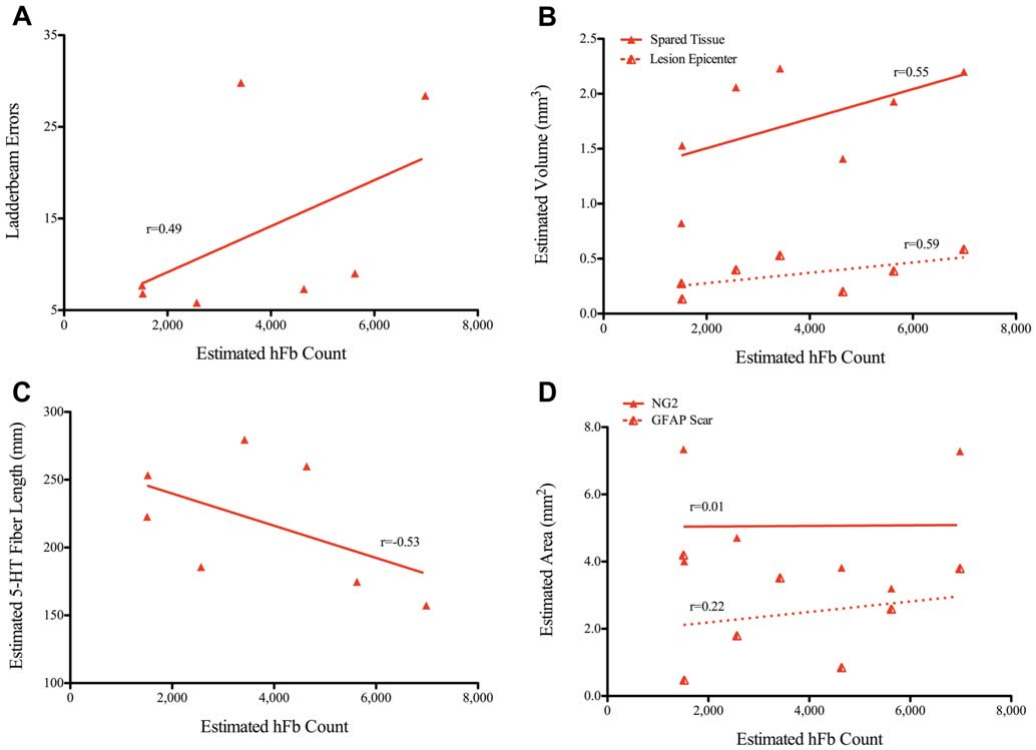
hFb engraftment does not correlate with behavioral or histological measures of recovery. A: In contrast to hCNS-SCns, linear regression analysis revealed a positive, but non-significant correlation between hFb engraftment and the number of errors made on the horizontal ladderbeam task *(Pearson r = 0.49, p = 0.26, 2-tailed t-test*). B–D: Linear regression analyses for the estimated number of hFb and other measures of host recovery revealed no significant correlations between cell engraftment and lesion volume (B) (*Pearson r: r = 0.59, p = 0.16, 2-tailed t-test*), volume of spared tissue (B) (*Pearson r: r = 0.55, p = 0.21, 2-tailed t-test*), serotonergic fiber sprouting (C) (*Pearson r: r = −0.53, p = 0.22, 2-tailed t-test*), NG2 area (D) (*Pearson r: r = 0.01, p = 0.98, 2-tailed t-test*), and area of the GFAP astroglial scar (D) (*Pearson r: r = 0.22, p = 0.63, 2-tailed t-test*).

Linear regression analysis revealed a significant negative correlation (*Pearson r: r = −0.78, p = 0.038, 2-tailed t-test*) between the estimated number of engrafted hCNS-SCns and the number of errors made on the horizontal ladderbeam (Fig. 11A). Interestingly, a positive but non-significant, correlation (*Pearson r: r = 0.49, p = 0.26, 2-tailed t-test*) was found between the estimated number of hFb and the number of errors made on the ladderbeam task (Fig. 12A), suggesting a trend for decreased behavioral recovery with an increase in hFb engraftment. These data extend and confirm our previous findings and suggest that hCNS-SCns, but not hFb engraftment is critical for locomotor recovery.

Analyses of other measures of host recovery revealed no significant correlations between cell engraftment and any parameters of host repair. No significant correlations were found between hCNS-SCns engraftment and lesion volume (*Pearson r*: *r = −0.70, p = 0.08, 2-tailed t-test)* as shown in Fig. 11B. In a contrasting yet still non-significant trend, no correlations between hFb engraftment and lesion volume (Fig. 12B) were detected (*Pearson r*: *r = 0.59, p = 0.16, 2-tailed t-test*). Fig. 11B demonstrates an absence of a significant correlation between hCNS-SCns engraftment and the volume of spared tissue (*Pearson r*: *r = −0.40, p = 0.37, 2-tailed t-test);* similarly, as shown in Fig. 12B, hFb engraftment did not correlate with tissue sparing (*Pearson r*: *r = 0.55, p = 0.21, 2-tailed t-test*). Correlation analysis of hCNS-SCns engraftment with serotonergic fiber length (Fig. 11C) demonstrated no significant correlations between these two measures (*Pearson r*: *r = 0.42, p = 0.34, 2-tailed t-test);* once again, in contrast to the positive yet non-significant trend for increased fiber sprouting in animals receiving hCNS-SCns, hFb engraftment (Fig. 12C) resulted in a negative but non-significant trend (*Pearson r*: *r = −0.53, p = 0.22, 2-tailed t-test*). Finally, no significant correlations between hCNS-SCns engraftment and NG2 area (*Pearson r*: *r = 0.16, p = 0.77, 2-tailed t-test),* and between hCNS-SCns engraftment and GFAP-positive astrogliosis (*Pearson r*: *r = −0.69, p = 0.09, 2-tailed t-test)* were observed (Fig. 11D), although the latter demonstrated a strong trend for reduction in astroglial scarring with an increase in hCNS-SCns engraftment. hFb engraftment also did not correlate with the area occupied by the NG2 proteoglycan (Fig. 12D) *(Pearson r*: *r = 0.01, p = 0.98, 2-tailed t-test*); as well, no significant correlations between hFb engraftment and GFAP-positive astrogliosis (*Pearson r*: *r = 0.22, p = 0.63, 2-tailed t-test*) were observed (Fig. 12D), but interestingly and again in contrast to hCNS-SCns, there was a strong yet non-significant trend for an increase in astroglial scarring with an increase in hFb number. Altogether, these data suggest that increased hCNS-SCns engraftment may result in reduced lesion size and astroglial scarring while hFb may exacerbate those measures of recovery. Correlative analysis for angiogenesis and hCNS-SCns/hFb engraftment could not be performed because the number of saline-treated animals with identifiable injury epicenters was too low (n = 4 for hCNS-SCns and n = 5 for hFb) to obtain reliable correlative results. Collectively, our findings do not provide conclusive evidence that an increase in hCNS-SCns number is more beneficial for recovery- a question currently under further investigation, but not within the scope of the current study. However, our data demonstrate that hCNS-SCns, but not hFb, survival and engraftment are directly related to a quantitative measure of behavioral recovery but not other measures of histological outcome. Notably, these results suggest that an increase in hCNS-SCns number does not have detrimental effects on lesion size, tissue sparing, regeneration, NG2 deposition, or astroglial scarring.

## Discussion

In our previous study, we reported locomotor recovery in NOD-*scid* mice receiving 60 kd contusion injuries and hCNS-SCns transplantation at 9dpi. Immuno-electron microscopy revealed evidence for human cell remyelination of mouse axons, the formation of putative human-mouse synapses, and ablation of human cells using DT abolished the observed recovery, suggesting that hCNS-SCns integrated within the host circuitry [1]. However, the hypothesis that hCNS-SCns altered the host microenvironment as an additional mechanism of recovery remained unexplored. In the present study, we tested this hypothesis and demonstrated that hCNS-SCns survived and migrated, and 80.5% of the transplanted cells were ablated after DT administration. No evidence for stereological and biochemical changes in the host microenvironment was found in animals transplanted with hCNS-SCns versus hFb or vehicle control mice. Notably, a negative and significant correlation was detected between hCNS-SCns (but not hFb) engraftment and the number of errors made on the horizontal ladderbeam task suggesting that survival/engraftment of a specific cell population (hCNS-SCns) is directly related to a quantitative measure of recovery. Human cell engraftment did not correlate with any other histological measures of recovery. Collectively, these data support the hypothesis that hCNS-SCns transplantation at 9dpi results in behavioral recovery via cell integration within the host circuitry.

### Engraftment and survival

A wide array of stem cell transplantation strategies has been tested for the potential to promote recovery in animal models of SCI. These include, but are not limited to, OECs [10], [45], [67], [73], bone marrow stromal cells [74], [75], Schwann cells [36], [44], genetically-modified fibroblasts [9], [11], [41], [76], embryonic-derived neural/glial stem cells [2], [4], [6], and fetal/adult NSCs [1], [3], [5], [7], [77]. While cell transplantation in many of these studies has been reported to result in improved recovery of function, engraftment and survival of the transplanted population have been, in most cases, either partially investigated or not addressed at all. Moreover, the number of animals with successful engraftment is often not reported. While it may be possible for trophic/neuroprotective effects of cell-mediated host repair to persist even if exogenous cells are lost in a delayed fashion, long-term survival of a transplanted cell population is critical in studies that report recovery of function associated with cell replacement and/or integration within the host circuitry, as in the case of our previous publication. In that regard, the NOD-*scid* mouse is the model of choice for xenograft experiments because of the ability to achieve successful engraftment in 100% of transplanted animals and long-term survival of xenografted cells. NOD-*scid* mice are widely used in human hematopoietic cell transplantation research [78]. We have previously extended the use of NOD-*scid* mice to include the CNS [18], [19] and observed high levels of engraftment and long-term hCNS-SCns survival in the neonatal brain. Thus, in order to achieve adequate control over host immunorejection in the injured rodent spinal cord, in this study, we chose the NOD-*scid* mouse model and report that 100% of transplanted animals exhibited engraftment of hCNS-SCns and hFb after a 9 day delayed transplantation.

An alternative approach for xenogeneic/allogeneic transplants is modulation of the immune response via administration of immunosuppressant drugs. However, the efficacy of long-term engraftment using pharmacological immunosuppressants is either not reported in most studies or appears to be limited. Only few studies transplanting NSCs into the injured spinal cord of immunosufficient animals receiving either cyclosporine or tacrolimus have attempted to numerically quantify total cell numbers at the time of sacrifice and report poor cell survival, ranging from 0.1%–37% [3], [5], [79], [80]. Critically, to our knowledge, only one study transplanting human NSCs into the spinal cord-injured immunodeficient athymic rat has reported a 375% increase (assessed by stereological methods) in total cell numbers at 6 months post-transplant [7]. Thus, it appears that pharmacological immunosuppression by means of cyclosporine or tacrolimus alone may be insufficient to obtain long-term survival. Additionally, in the clinical setting, neurotoxicity is one of the most widely reported side effects of both of these immunosuppressant drugs [81], [82]. Similarly, administration of immunosuppressant agents may cause toxicity in animals resulting in illness and poor behavioral performance. Finally, the effects of immunosuppressant drugs on cell fate and mechanisms of recovery remain largely unexplored.

Altogether, these data suggest that the use of a constitutively immunodeficient model avoids the possible confounds of immunosuppressant drugs while providing an adequate transplantation environment where empirical hypotheses about cell survival and mechanisms of recovery could be tested. In the current study, stereological quantification of human cells transplanted into the immunodefiicent microenvironment revealed: 1) successful engraftment of human cells in 100% of animals receiving either hCNS-SCns or hFb; 2) limited proliferation of hCNS-SCns (194% of the number originally transplanted) and poor engraftment/survival of hFb (7.5% of the number originally transplanted) at 17 weeks post-transplant.

### Factors influencing cell-mediated potential for repair of neurotrauma

Multiple mechanisms by which engrafted stem cells could either directly integrate with the host or indirectly affect the host microenvironment following CNS injury have been proposed. Yet, few studies transplanting stem cell populations have thoroughly addressed the mechanisms of recovery following engraftment. We have previously shown evidence for cell integration after 9 day delayed hCNS-SCns transplantation into NOD-*scid* mice. In the present study, further analysis of transplanted animals demonstrated no changes in the host microenvironment as measured by lesion size and tissue sparing, serotonergic regeneration/sprouting, NG2 deposition, glial scarring, and angiogenesis as measured by stereology. Additionally, biochemical protein analysis supplemented our stereological quantification and demonstrated no changes in protein expression levels of Fibronectin, NG2, Versican, GFAP, and PE-CAM1 at 2 weeks post-transplant. Collectively, the stereological and biochemical analyses suggest that transplantation of hCNS-SCns into contusion injured NOD-*scid* mice at 9dpi does not result in changes within the host microenvironment. While it is possible that other measures of recovery not assessed in the current study have undergone change as a result of hCNS-SCns transplantation, we drew on the current literature in order to select the most likely candidates for host modification. We expect that a number of factors could influence the potential repair mechanisms of the transplanted cells.

One variable that may influence the capacity of cells for neurorepair is the *nature* of the transplanted cell population. The source of transplanted NSCs (i.e. murine vs. human, ES vs. fetal) could affect the interpretation of SCI studies addressing mechanisms of recovery *in vivo*. Diversity in the source of original cell populations, methods of isolation, and preparation of cells could affect cell fate and the resultant effect of the cells on the host microenvironment. In the current study, we use a human NSC population that is prospectively isolated based on FACS to identify a CD133^+^ and CD24^−/lo^ population. This process theoretically selects for a NSC-enriched population; accordingly, these cells exhibit approximately a 2000-fold enhancement in neurosphere initiating capacity [18]. It is unknown whether the cell intrinsic-properties of NSCs isolated by this or similar methods differ from other NSC populations; however, it is possible that selection for specific markers could alter either the response of the cells to the microenvironment or, conversely, the effect of the microenvironment on the cells.

Another variable that may affect the potential mechanisms of recovery is the *nurture* of the cell population. Both the heterogeneity and differentiation capabilities of NSCs could be affected by the method used to derive and culture the cells as either a monolayer [7], [83]–[86] or as neurospheres [87] prior to transplantation. When cultured under conditions that do not promote differentiation, NSCs grown as monolayers can retain their multipotent capacity and give rise to neural precursor/progenitor populations [85], [86], [88]. When expanded as neurospheres, NSCs represent a heterogeneous population of both multipotent neural stem cells and more restricted progenitors [89]. It is possible that cells grown as monolayers versus neurospheres, as is the case in the present study, would not behave in the same manner in an *in vivo* setting and thus affect the mechanisms of recovery.

Finally, the *niche* into which the cells are transplanted could override the intrinsic properties of transplanted cells and affect mechanisms of recovery. The niche can be defined by the animal model, the injury paradigm, location of the transplant, presence or absence of immunosuppression, and time of transplantation. In the present study, hCNS-SCns were transplanted at 9dpi into the intact spinal cord parenchyma of contused NOD-*scid* mice. As discussed earlier, our animal model was selected to obtain successful cell engraftment and to avoid confounds of immunosuppressant agents, which are often used in SCI transplantation studies.

Another aspect of the niche is the type of injury. In this study, we used the clinically relevant experimental model of contusion injury. According to the National Spinal Cord Injury Statistical Center, motor vehicle crashes, the most common type of contusion injuries, account for 42% of reported SCI cases in humans. In animal models, contusion injuries are induced by exposing the spinal cord to mechanical trauma while the dura is maintained intact [72], [90]–[92]. Contusion injury results in neuronal apoptosis as well as oligodendroglial cell death rostral and caudal to the lesion site beginning at 6 hours and extending to at least 3 weeks post-injury [38], [93]. Further, contusion injury has been shown to preferentially target the large caliber axons [94], suggesting neuronal damage, oligodendroglial loss, and subsequent demyelination post-trauma. Spinal cord pathology following contusion injury is also accompanied by a prolonged secondary inflammatory response [95] that includes the activation of resident microglia and recruitment of immune cells, such as macrophages, rostrocuadal to the injury epicenter, and especially in the white matter [96], [97], suggesting phagocytosis of myelin debris. In contrast, transection of the spinal cord results in a narrow zone of primary tissue damage that is followed by a focal secondary pathological and inflammatory response [97]. Cell death following transection models of injury is thought to affect neurons and glia indiscriminately and morphological analyses of dying cells in the transected cord suggests a necrotic mechanism [98]. Collectively, these data suggest that the experimental injury paradigm is a critical factor in defining the host niche post-trauma.

In the present study, hCNS-SCns were transplanted into the intact parenchyma of the spinal cord near the lesion site but not directly into the injury epicenter. This was done for two reasons: 1) While rats and most humans form a cavity at the lesion epicenter after SCI, mice develop a fibronectin-rich matrix at the injury site [99]. This compact meshwork of cells and connective tissue may limit the injection volume for transplantation. 2) The initial peak of inflammatory CD11b/ED1-positive cells in the rodent injury epicenter occurs at 7–14dpi [37], [95]. The presence of this phagocytic cell population at the injury site suggests that the epicenter may not be a favorable environment for cell survival, and the effects of myeloid cells on fate potential have not been thoroughly investigated. Further, proinflammatory cytokines, such as IL-1β, peak at 12 hrs post-SCI but are found for up to at least 28 dpi [100]. Other cytokines such as TNF-α and IFN-γ have also been detected at elevated levels in the injury epicenter at 7dpi [101]. Collectively, these data suggest that at 9dpi, the injury epicenter may not afford optimal cell survival. Thus, we transplanted hCNS-SCns into the relatively normal parenchyma adjacent to the lesion site.

Finally, most transplantation studies after SCI are performed within a “therapeutic window” of 7–14dpi when the potential for cell replacement is thought to be optimal [102]. However, cell replacement is not the only means by which transplanted cells could promote recovery of function. Several studies have suggested that acute (0–5dpi) transplantation of NSCs and neural progenitor cell populations can promote recovery by alternative mechanisms, e.g. via modification of the host niche [12], [13], [15]. In the present study, we tested this hypothesis for hCNS-SCns engraftment at a slightly more delayed timepoint of 9dpi and found no changes in the host microenvironment as a result of hCNS-SCns transplantation. It is possible that shortening the delay between SCI and cell transplantation may drive the transplanted cells to serve a more trophic role and alter the host microenvironment. Alternatively, the mechanisms mediating recovery of function may be cell population-specific.

Finally, contusion/compression injuries comprise the majority of human spinal cord injuries, and most injuries of this type are categorized as anatomically incomplete, suggesting the presence of spared host axons and circuitry post-injury [103]–[105]. Contusion SCI models in rodents recapitulate many features of clinical contusion/compression injuries, suggesting that understanding the mechanisms of recovery, i.e. integration versus stimulation of host-mediated repair, will yield insight into strategies for the treatment of clinical SCI, and the appropriate timing for potential cellular therapeutic interventions.
